# Prognostic and Risk Factors in Patients with Locally Advanced Cutaneous Squamous Cell Carcinoma of the Trunk and Extremities

**DOI:** 10.1155/2011/420796

**Published:** 2011-05-19

**Authors:** de Lima Vazquez Vinicius, Cristovam Scapulatempo, Natalia Martins Perpetuo, Faheez Mohamed, Teóclito Sachetto de Carvalho, Antônio Talvane Torres de Oliveira, José Getúlio Martins Segalla, André Lopes Carvalho

**Affiliations:** ^1^Department of Surgery and Pathology, Hospital de Câncer de Barretos, Rua Antenor Duarte Villela, 1331-Bairro Paulo Prata, 14784-400 Barretos, SP, Brazil; ^2^Division of Surgery, Basingstoke and North Hampshire NHS Foundation Trust, ldermaston Road Basingstoke, Hampshire RG24 9NA, UK; ^3^Department of Clinical Oncology, Hospital Amaral Carvalho, R Dona Silveria, 150 17203-570 Jaú SP, Brazil

## Abstract

55 patients with advanced cutaneous squamous cell carcinoma (CSCC) of the trunk and extremities were studied. *A Tissue Microarray* was constructed using immunohistochemistry to quantify expression of the HER family, E-cadherins, and podoplanin. Clinical and histopathological factors related to lymph node metastasis and prognosis were also established. Primary tumor positivity was 25.5% for EGFR, 87.3% for HER-3, and 48.1% for HER-4. Metastases were positive for EGFR in 41.7%, for HER-3 in 83.3%, and HER-4 in 43.5%. HER-2 was negative in all samples. Membrane E-cadherin and cytoplasmic E-cadherin were positive in 47.3% and 30.2% of primary tumors and 45.5% and 27.3% of metastases, respectively. Podoplanin was positive in 41.8% of primary tumors and 41.7% of metastases. Intratumoral lymphocytic infiltrate was associated with lymph node metastasis. Patients with T3 tumors had better cancer-specific survival (CSS) than those with T4 tumors; patients with no lymph node involvement had better CSS than patients with N1 tumors. Undifferentiated tumors and hyperexpression of podoplanin were negative prognostic indicators on multivariate analysis.

## 1. Introduction

“Locally advanced cutaneous squamous cell carcinoma of the trunk and extremities has a poor prognosis. This study identified prognostic factors including podoplanin, a novel molecular marker.”

Cutaneous squamous cell carcinoma (CSCC) has a high incidence worldwide particularly in the sun exposed skin of Caucasians [1–5]. The majority of cases are readily treatable by simple excision or radiotherapy with a good chance of achieving cure. However, locally advanced tumors may present with local recurrence, lymph node or distant metastasis [6–12]. Unlike head and neck tumors, where the presence of lymph node metastases and disease progression are more common, Prognostic factors for advanced tumors of the trunk and extremities are not well established. Clinical and epidemiologic factors are poorly understood with only a few reports in the literature [10, 11, 13–15]. Knowledge of the role of molecular markers in tumor progression and metastasis is limited. The tyrosine kinases Human Epidermal Receptor (HER) family (Epidermal Growth Factor Receptor (EGFR), HER-2, HER-3, and HER-4) are transmembrane glycoproteins related to cell proliferation, differentiation, and apoptosis [16]. Altered expression of the HER family is associated with several epithelial tumors such as breast carcinoma and esophageal squamous cell carcinoma [17–20]. Small studies have also shown altered HER expression in localized squamous cell carcinoma when compared to normal skin [21–24]. HER expression in advanced CSCC of the trunk and extremities is not well studied and may be related to prognosis allowing the use of targeted therapies that block the HER pathway.

E-cadherin is a transmembrane glycoprotein, and it is a mediator of calcium-dependent cell-cell adhesion in normal cells [25]. Reduced cell-cell adhesiveness is considered important in both early and late carcinogenis [25, 26]. High E-cadherin expression in cell cytoplasm and low expression in the cell membrane are associated with tumor aggressiveness in different cancers, (i.e., lung cancer).

 Podoplanin is a membrane protein found on lymphatic vessel endothelium. Its function is poorly understood although it may govern endothelial motility, and its absence in animal studies is associated with lymphedema and malformation of lymphatic vessels [27]. The aim of this study was to determine the expression of markers such as the HER family, E-cadherin, and Podoplanin in a consecutive series of locally advanced CSCC of the trunk and extremities and to define clinical, pathological, and molecular factors related to lymph node metastasis and survival.

## 2. Methods

A retrospective study of patients with locally advanced (American Joint Committee on Cancer staging T3 and T4 ) CSCC of the trunk and extremities admitted to two cancer institutions in Brazil (Barretos Cancer Hospital and Amaral Carvalho Hospital) between 1997 and 2006 was performed. Only those patients with tumor paraffin blocks available for analysis were included. Patients with tumor infiltration of the head and neck or genital area and those with a previous cancer diagnosis other than cutaneous basal cell carcinoma were excluded. This was to avoid difficulties in identifying origin of metastasis and cause of death. 55 consecutive patients admitted and treated from October 1997 to March 2006 with a pathologic diagnosis of squamous cell carcinoma were evaluated. Patients had to have stage T3 (tumor >5 cm) or T4 (invasive of deep extradermal structures) tumors according to the 2002 American Joint Committee on Cancer (AJCC) staging system. Institutional Review Board approval was obtained and all clinical information retrospectively collected from medical records. 

### 2.1. Demographic and Clinical Characteristics

Demographic and clinical variables assessed included age, gender, ethnicity, previous chronic skin lesions (burns, scars, varicose ulcers and others) at the site of the tumor, patient residence (rural or urban), anatomic site, and treatment. Patterns of lymph node metastases, recurrence, and survival outcomes were also recorded.

 Lymph node metastasis was classified as follow: N0: patients with no evidence of lymph node metastasis at presentation; N1: patients with lymph node metastasis at presentation. We considered lymph node metastasis at presentation (N1) or recurrence as the endpoint for risk of lymph node metastasis. The endpoint for survival was death from cancer. Only clinically involved lymph nodes were removed and no elective or sentinel node dissections were performed. 

### 2.2. Pathology

55 primary tumors and 22 lymph node metastases were available for pathological review by two pathologists. The pathological variables analyzed were number of mitosis/mm^2^, deepest tumor diameter (Breslow depth), tumor grade I to III as previously described [28], perineural or perivascular infiltration, and intratumoral and peritumoral lymphocytic infiltration. Any lymphocytic infiltration was quantified as positive. Breslow depth was available in 44 cases. 

### 2.3. Tissue Micro array

After pathological review, the most representative tumor area in the paraffin block was selected for creation of a tissue microarray (TMA). Both primary tumors and lymph node metastases were selected. A Manual Tissue Arrayer I, (Beecher Instruments, EUA) was used to obtain two cylinders of 1.0 millimeter in diameter from each paraffin bloc. These were implanted into the receptor paraffin block (TMA). Fifty slides were obtained and numbered from the TMA. For sample quality analysis slide numbers 1, 25, and 50 were stained with hematoxilin and eosin, and the most representative was chosen, and the other slides studied were subsequent to this. 

### 2.4. Immunohistochemistry

Deparaffinization of the sections was done with xylene for 15 minutes at 60°C, followed by 15 minutes at room temperature. The sections were then washed 3 times for 30 seconds with 100%, 95%, 80%, and 70% ethanol before washing in water. Endogenous peroxidase was blocked by incubating the sections in 6% hydrogen peroxide in methanol. The sections were then washed with phosphate buffered saline (PBS) 10 mM pH 7.4 for 5 minutes. Incubation followed, as described by Hsu and Raine [29] with the specific antibody diluted in PBS with 1.0% bovin serum albumin (Sigma USA) and 0.1% NaN_3_ for 30 minutes at 37°C and for 16 hours at 4°C.

For EGFR the H11 clone (DAKO) was used, diluted 1 : 100 in an autoclave with EDTA at pH 8.0. The following HER family polyclonal antigens were used all with citrate at pH 6.0: HER-2 (DAKO) diluted 11500 in a moist chamber, HER-3 (Neomarkers) 1 : 100 in an autoclave, (HER-4) (Neomarkers) 1 : 300 in an autoclave. The E-cadherin NCH-38 (DAKO) monoclonal antigen was diluted 1 : 600 in a moist chamber with EDTA/TRIS at pH 9.0. The Podoplanin D2–40 clone (DAKO) was used at a dilution of 1 : 200 in a moist chamber with EDTA/TRIS at pH 9.0. After incubation they were washed with PBS 3 times for 5 minutes each and then antigen amplification was performed. After amplification they were again washed with PBS 3 times for 5 minutes each. Reactions were visualized with 0.6 mg/mL 3′-3′ diaminobencidine tetrahydrochloride and 0.06% hydrogen peroxide in a 1% PBS solution for 5 minutes at 37°C. The final reaction was a brown color deposit in the cell area where the antigen-antibody reaction had occurred. 

### 2.5. Immunohistochemistry Expressions

The EGFR, HER-3, and HER-4 expressions were evaluated semiquantitatively according to the method described by Lager et al. [30]. Areas with more intense reaction were selected, and the intensity of cytoplasm and/or membrane reaction was classified as 0 negative; + weak; ++ moderate; +++ intense. For the study, tumors classified as 0 or + were considered negative and tumors classified as ++ or +++ were considered positive. 

HER-2 expression was evaluated semiquantitatively according to the intensity of reaction in the cytoplasmatic membrane. Negative or weak reaction in less than 10% of cells was classified as “0”, weak reaction in more than 10% as “+”, moderate reaction in more than 10% as “++”, and strong reaction in more than 10% as “+++” positivity. For the analysis, 0 and + were considered negative and ++ and +++ positive [31].

 Cytoplasmic and membranous E-cadherin immunoexpressions were semiquantitatively evaluated as negative if the reaction occurred in up to 50% of cells and positive if occurred in more than 50% of cells. 

 The podoplanin (D2–40) immunoreactivity was semiquantitatively evaluated as described by Padgett et al. [32]: negative: no reactivity or weak reaction independently of the number of cells or moderate/strong reaction in up to 10% of cells; positive: moderate or intense immunoreaction in more than 10% of cells. 

### 2.6. Statistics

To analyze the association between clinical variables and lymph node metastasis the chi square, Fisher exact, and *t*-test were used. Specific Cancer Survival (SCS) was also studied, and curves were constructed using the Kaplan-Meier method and compared using the univariate log-rank test. All tests were two sided, and a *P*-value of ≤.05 was considered statistically significant. Simultaneous prognostic effect of various factors was determined in a multivariate analysis by use of the Cox proportional-hazards regression model with a covariate of primary interest and adjustment covariates.

## 3. Results

### 3.1. Demographic, Clinical, and Pathological Descriptive Characteristics

55 patients with a Mean age of 63 years (Range 30–91) were included in the study. 

The median number of mitosis/mm^2^ was 3 and median Breslow depth was 8 mm. Tumor characteristics are shown in Table 1. 

### 3.2. Immunohistochemistry

EGFR positivity was 25.5% in the primary tumor and 41.7% in the metastases. HER-2 was negative in all samples. HER-3 and HER-4 positivity was 87.3% and 48.1% in the primary tumor and 83.3% and 43.5% in the metastases, respectively. Membrane E-cadherin positivity was 47.3% in the primary tumor and 27.3% in the metastases. Primary tumor cytoplasmic E-cadherin was positive in 30.2% and 45.5% in the metastasis. The E-cadherin membrane/cytoplasmic ratio was 1.56 in the primary tumor and 0.60 in the metastases. Podoplanin positivity was 41.8% in primary tumor and 41.7% in metastases. 

### 3.3. Risk of Lymph Node Metastasis

Intratumoral lymphocytic infiltrate was the only prognosticator of lymph node metastasis (92% versus 66.6%; *p* = 0.046) (Table 2). 

### 3.4. Survival

The mean and median followup was 9.6 (SD 25.0) and 25.0 months, respectively. At last followup, 19 patients were alive with no evidence of disease (34.5%), one was alive with disease (1.8%), 19 were dead of disease (34.5%), 9 dead from other causes (16.4%), and 7 lost to followup (12.7%). Those lost to followup had a mean and median followup of 24.5 (SD 21.8) and 22.1 months respectively. Only two patients were followed up for less than one year and three patients for less than 22 months. For the 11 patients that presented with lymph node metastasis during the followup, median time to occurrence was 13.08 months. 

**Figure 1:**
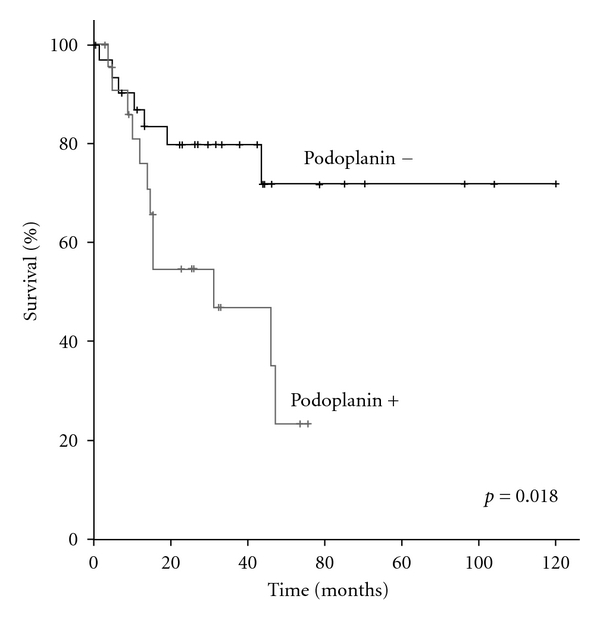


The overall five years cancer-specific survival (CSS) was 49.7%. For patients with T3 tumors 5-year CSS was 67.6%, and no patients with T4 tumors were alive at 5 years (*p* = 0  .001). Patients with no lymph node metastases had a 5-year CSS of 63.3%, with no 5 year survivors in patients with lymph node metastases (*p* = 0.004). Gender, race, ambient, anatomic location, location of metastasis, and presence of previous nononcologic lesions did not affect survival (Table 3). There was no difference in 5-year CSS between patients with primary tumors with up to 3 mitosis/mm^2^ and those with more than 3 mitosis (49.7% versus 47.2%, *p* = 0  .375). No significant difference in 5-year CSS was seen for Breslow depth, peritumoral lymphocytic infiltrate, vascular, and perineural infiltration. The only histological variable that had a significant impact on survival was the tumor grade. Patients with grade I lesions had a 5-year CSS of 82.2% compared with 23.8% in patients with grade II and III tumors (*p* = 0  .010). Podoplanin negative patients had a higher 5 years (Figure 1, Table 4). In metastatic tumors, HER-4 negativity resulted in a 3-year CSS of 66.7% versus 37.5% in HER-4 positive patients (*p* = 0  .038). Clinical stage and Podoplanin positivity were independent prognostic factors on both univariate and multivariate analysis (Table 5). 

## 4. Discussion

### 4.1. Demographics and Clinical Characteristics

Previous nonskin cancer lesions at the site of disease were seen in 23% of patients. Although not associated with lymph node metastasis or poor prognosis, unlike previous reports [6], the presence of noncancerous skin lesions may result in locally advanced disease due to mis- or delayed diagnosis. The T stage of tumor strongly influenced survival but not incidence of lymph node metastasis. The high number of N1 patients (25.5%) has been previously described in an identical setting [10], and may be associated with the two Hospitals involved being tertiary referral oncology centers. 

### 4.2. Pathological Characteristics

Intratumoral lymphocytic infiltration was associated with lymph node metastasis. The inflammatory response may lead to greater tumor antigen exposure in the metastatic lymph node. Tumor thickness (Breslow) had no impact on lymph node metastasis and did not influence survival. Breslow depth may correlate with lymph node metastasis and survival in less advanced tumors, but in this study median tumor thickness was high which may have weakened any association. Low-grade tumors were associated with prolonged survival confirming the aggressive nature of undifferentiated tumors. 

### 4.3. Molecular Markers

Unlike squamous cell carcinoma of the head and neck or esophagus, EGFR had no influence on prognosis. It is possible that altered EGFR expression may be associated with local recurrence, which is more frequently life threatening at other sites. HER-2 was negative in all samples and may play little part in CSCC progression as found in squamous cell carcinomas from other sites. High HER-4 expression in lymph node metastases was associated with poor prognosis suggesting a role in progression of CSCC of the trunk and extremities. It is possible that altered HER-4 expression occurs late and is present only in metastases. The altered coexpression of the HER family may play a role (i.e., EGFR/HER-4, HER-3/HER-4, and EGFR/HER-3/HER-4) but the small number of cases in this study meant this could not be analyzed. E-cadherin expression had no significant association with lymph node metastasis or survival, but the expression ratio between membrane and cytoplasm was lower in the metastasis, suggesting accumulation with a loss of function. This may be due to a mutated E-cadherin resulting in cytoplasmic accumulation with a loss of cell adhesion and disease progression [33, 34]. Some studies have looked at the membrane expression of E-cadherin in cutaneous squamous cell carcinoma compared with normal skin, local tumors, and metastasis, suggesting a progressive loss of expression [35–37]. 

Podoplanin expression was not associated with the presence of lymph node metastasis, but was a prognosticator of reduced survival indicating a locally aggressive tumor, with survival impact. Altered expression of podoplanin is associated with mesothelioma, squamous cell carcinoma of oral mucosa, and germ cell tumors, suggesting that podoplanin may influence invasive and proliferative activity [38–40]. As CSCC metastases occur preferentially via lymphatic vessels, podoplanin expression may be associated with disease progression. Hyperexpression has been related to undifferentiated skin tumors, but its impact on prognosis and metastasis has not been established [41]. Podoplanin is a possible target for development of novel therapies, and its expression has to be studied in other settings to completely understand its role in cancer development and progression.

Patients with advanced CSCC of trunk and extremities with poor prognostic factors such as undifferentiated, T4, N1 tumors, high podoplanin expression in the primary tumor, or high HER-4 expression in the lymph node metastasis may be candidates for new more aggressive modalities of treatment. Further studies using these molecular markers are needed to help refine treatment of CSCC of the trunk and extremities. 

## Figures and Tables

**Table 1:** Demographic, clinical, therapeutic, and pathological characteristics of patients with locally advanced cutaneous squamous cell carcinoma of trunk and extremities.

Characteristics	*n *(%)	Characteristics	*n *(%)
Gender		Lymph node metastasis location	
Male	32 (58.2)	Axila	15 (60.0)
Female	23 (41.8)	Groin	10 (40.0)
Race		Lymph node metastasis treatment	
Caucasian	49 (89.1)	Linphadenectomy	28 (93.3)
African	6 (10.9)	No treatment	2 (6.7)
Residence		Local or lymph node recurrence after lymphadenectomy	
Rural	9 (16.4)	No	13 (46.4)
Urban	46 (83.6)	Local	8 (28.6)
		Lymph node	7 (25.0)
Chronic sun exposure		Distant metastasis	
Yes	27 (49.1)	No	58 (92.1)
No	12 (21.8)	Cutaneous	1 (1.6)
n.a.	16 (29.1)	Visceral	4 (6.3)
Anatomical localization		Lymph node metastasis treatment	
Lower extremities	22 (40.0)	Linphadenectomy	24 (96.0)
Upper extremities	23 (41.8)	No treatment	1 ( 4.0)
Trunk	10 (18.2)		
Non-cancer previous lesion		Tumor grade	
Yes	13 (23.6)	I	25 (45.5)
No	42 (76.4)	II	27 (49.0)
		III	3 (5.5)
T classification		Intratumoral lymphocitic infiltrate	
T3	33 (60.0)	Negative	12 (21.8)
T4	22 (40.0)	Positive	43 (78.2)
N classification		Peritumoral lymphocitic infiltrate	
N0	41 (74.5)	Negative	9 (16.4)
N1	14 (25.5)	Positive	46 (83.6)
Clinical stage		Vascular infiltrate	
II	27 (49.1)	Negative	48 (87.3)
III	28 (50.9)	Positive	7 (12.7)
Treatment of primary tumor		Perineural infiltrate	
surgery		Negative	54 (98.2)
Local resection with primary closure	1 ( 1.9)	Positive	1 (1.8)
Local resection with reconstruction	18 (32.7)		
Amputation/disarticulation	21 (38.2)		
Local resection with open wound	5 ( 9.1)		
Radiation therapy	8 (14.5)		
No treatment	2 (3.6)		

n.a.: not available.

**Table 2:** Univariate analysis of risk factors for lymph node metastasis in patients with locally advanced cutaneous squamous cell carcinoma of trunk and extremities.

Variables	+ Lymph node metastasis *n* (%)	− Lymph node metastasis *n* (%)	*p*
*Clinical*			
Gender			
Male	16 (64.0%)	16 (53.3%)	0.584
Female	9 (36.0%)	14 (46.7%)	
Race			
Caucasian	22 (88.0%)	27 (90.0%)	1.000
African	3 (12.0%)	3 (10.0%)	
Ambient			
Rural	4 (16.0%)	5 (16.7%)	1.000
Urban	21 (84.0%)	25 (83.3%)	
Chronic Sun exposure			
Yes	14 (66.7%)	13 (72.2%)	0.742
No	7 (33.3%)	5 (27.8%)	
Anatomic location of primary tumor			
Lower extremities	8 (32.0%)	14 (46.7%)	0.151
Upper extremities	14 (56.0%)	9 (30.0%)	
Trunk	3 (12.0%)	7 (23.3%)	
Previous non neoplasic lesion			
Yes	7 (72.0%)	6 (20.0%)	0.537
No	18 (28.0%)	24 (80.0%)	
TNM Classification			
T3	13 (52.0%)	20 (66.7%)	0.286
T4	12 (48.0%)	10 (33.3%)	
Tumor length*			
Breslow 0–8 mm	14 (56.0%)	8 (44.5%)	0.533
Breslow >8 mm	10 (44.0%)	10 (55.5%)	
Mitosis/mm^2^			
0–3	22 (44.9%)	27 (55.1%)	0.573
>3	3 (50.0%)	3 (50.0%)	
Tumor grade			
I	11 (44.0%)	14 (46.6%)	1.000
II-III	14 (56.0%)	16 (53.4%)	
Intratumoral lymphocitic infiltrate			
Negative	**2 (8.0%)**	**10 (33.4%)**	**0.046**
Positive	**23 (92.0%)**	**20 (66.6%)**	
Peritumoral lymphocitic infiltrate			
Negative	2 (8.0%)	7 (23.4%)	0.160
Positive	23 (92.0%)	23 (76.6%)	
Vascular infiltrate			
Negative	20 (80.0%)	28 (93.3%)	0.226
Positive	5 (20.0%)	2 ( 6.7%)	
Perineural infiltrate			
Negative	24 (96.0%)	30 (100.0%)	0.455
Positive	1 (4.0%)	0 (0.0%)	
*Tumor markers*			
EGFR			
Negative	17 (68.0%)	24 (80.0%)	0.363
Positive	8 (32.0%)	6 (20.0%)	
HER-2			
Negative	25 (100%)	30 (100%)	Not calculated
Positive	0	0	
HER-3			
Negative	1 (4.0%)	6 (20.0%)	0.112
Positive	24 (96.0%)	24 (80.0%)	
HER-4			
Negative	16 (65.0%)	12 (41.4%)	0.083
Positive	9 (35.0%)	17 (59.6%)	
Membrane E-cadherin			
Negative	12 (54.5%)	14 (46.7%)	0.779
Positive	10 (45.5%)	16 (53.3%)	
Cytoplasm E-cadherin			
Negative	17 (73.9%)	20 (46.6%)	0.764
Positive	6 (26.1%)	10 (63.4%)	
Podoplanin			
Negative	13 (52.0%)	19 (63.3%)	0.425
Positive	12 (48.0%)	11 (36.7%)	

*Only 42 cases.

**Table 3:** Comparative specific cancer survival rates according to clinical variables in patients with locally advanced cutaneous squamous cell carcinoma of trunk and extremities.

Variable	Five years survival (%)	S.E.	*p*
Gender			
Male	42.9	11.3	0.172
Female	64.8	16.0	
Race			
Caucasian	50.2	10.8	0.443
African	44.4	22.2	
Ambient			
Rural	71.4	17.1	0.598
Urban	46.0	11.1	
Chronic Sun exposure			
Yes	47.5	11.5	0.972
No	00.0	00.0	
Anatomic location of primary tumor			
Lower extremities	61.0	15.5	0.441
Upper extremities	38.7	15.1	
Trunk	55.6	16.6	
Lymph node metastasis location			
Axila	28.1	15.6	0.858
Groin	00.0	00.0	
Previous non neoplasic lesion			
Yes	39.1	18.6	0.221
No	54.3	11.2	
TNM Classification			
T3	**67.6**	**10.8**	**0.001**
T4	**00.0**	**00.0**	
Lymph node status			
N0	**63.3**	**10.9**	**0.004**
N1	**00.0**	**16.8**	
Clinical stage			
II	**84.3**	**7.2**	**<0.001**
III	**00.0**	**00.0**	

**Table 4:** Comparative specific cancer survival rates according to immunohistochemical primary tumor expression in patients with locally advanced cutaneous squamous cell carcinoma of trunk and extremities.

Variable	5-year survival (%)	S.E.	*p*
EGFR			
Negative	52.1	10.8	0.592
Positive	34.6	25.3	
HER-2			
Negative		All cases negative	
Positive			
HER-3			
Negative	0.0	0.0	0.231
Positive	52.5	10.3	
HER-4			
Negative	49.6	12.4	0.632
Positive	44.0	17.0	
Membrane E-cadherin			
Negative	38.3	12.7	0.112
Positive	65.5	13.9	
Cytoplasm E-cadherin			
Negative	35.6	12.5	0.296
Positive	72.5	11.8	
Podoplanin			
Negative	**71.9**	**10.1**	**0.018**
Positive	**23.5**	**13.2**	

**Table 5:** Multivariate Cox regression model for specific cancer survival in patients with locally advanced cutaneous squamous cell carcinoma of trunk and extremities.

Variable	HR	95% CI	*p*
Clinical stage			
II	1		0.003
III	5.903	1.861–18.728	
Podoplanin			
Negative	1		0.050
Positive	2.839	1.011–8.128	
Age	1.010	0.978–1.044	0.543
Treatment			
Surgery	1		0.978
Radiotherapy	0.981	0.254–3.785	
